# The effect of *Kappaphycus alvarezii* fraction on plasma glucose, Advanced Glycation End-products formation, and renal RAGE gene expression

**DOI:** 10.1016/j.heliyon.2021.e05978

**Published:** 2021-01-19

**Authors:** Evy Yulianti, Sunarti, Mae Sri Hartati Wahyuningsih

**Affiliations:** a Department of Biology Education, Faculty of Mathematics and Science, Universitas Negeri Yogyakarta, Yogyakarta, Indonesia; b Doctoral Candidate at Department of Pharmacology and Therapy, Faculty of Medicine, Public Health and Nursing, Universitas Gadjah Mada, Yogyakarta, Indonesia; c Department of Biochemistry, Faculty of Medicine, Public Health and Nursing, Universitas Gadjah Mada, Yogyakarta, Indonesia; d Department of Pharmacology and Therapy, Faculty of Medicine, Public Health and Nursing, Universitas Gadjah Mada, Yogyakarta, Indonesia; e Herbal Medical Center, Faculty of Medicine, Public Health, and Nursing, Universitas Gadjah Mada, Yogyakarta, Indonesia

**Keywords:** *Kappaphycus alvarezii*, Diabetics, Glycation, IC_50_, Glucose, Glycated albumin, Nε- (carboxymethyl) lysine, RAGE

## Abstract

**Background:**

*Kappaphycus alvarezii* (Doty) Doty ex P.C.Silva is a red algae with antioxidant and antiglycation activities. Algae still have not been widely used for treating diabetes, especially to prevent complications. The purpose of this study was to examine the effect of active fractions from *Kappaphycus alvarezii* on plasma glucose level, glycation process and renal RAGE gene expression.

**Methods:**

This study used bioassay-guided fractionation, consisting of three stages: extraction, partition, and fractionation. These processes were monitored with Thin Layer Chromatography and the BSA-Glucose method to select the best extract with antiglycation activity (calculated as the percentage of inhibition and IC_50_). The selected active fraction from four fractions was further used for *in vivo* study, which was conducted with hyperglycemic Wistar male rats. Plasma glucose level was measured using GOD-PAP methods, while plasma glycated albumin (GA) and Nε- (carboxymethyl) lysine (CML) levels were measured using ELISA. Renal RAGE gene expression was analyzed using qPCR.

**Results:**

Fraction II was selected as the active fraction of *Kappaphycus alvarezii* showing antiglycation activity with the highest percentage of inhibition and the lowest IC_50._ This fraction significantly reduced plasma GA and CML levels, but it did not significantly reduce plasma glucose level. Furthermore, renal RAGE gene expression was lower in the diabetic rat group treated with this active fraction compared to the untreated group.

**Conclusions:**

This study successfully identified an active fraction of *Kappaphycus alvarezii* with antiglycation activity to reduce plasma GA and CML levels as well as renal RAGE gene expression. Therefore, this fraction could be developed as a potential candidate for treating diabetes.

## 1 Introduction

Glycation, also known as the Maillard reaction, is a slow nonenzymatic reaction, starting by the attachment of glucose or its derivatives to a protein amine group and the formation of a Schiff base, followed by the Amadori rearrangement to form a stable fructosamine residue (ketoamine) (Abate et al., 2015). Further oxidation and rearrangement of the initial glycation product irreversibly result in Advanced Glycation End-products (AGEs). This process is often associated with an oxidative phenomenon called "glycoxidation", which occurs when an oxidative reaction affects the initial glycation product (Baraka-Vidot et al., 2014). During the aging process, AGEs are formed and accumulate in blood vessel walls. These processes are increased in patients with hyperglycemia and also diabetes. The accumulation of AGEs in blood vessel walls and different organs leads to various disorders, particularly metabolic disorders, such as diabetes and obesity (Papagrigoraki et al., 2017; Ott et al., 2014). In these disorders, excessive AGEs accumulation can cause further tissue damage and is associated with diabetic clinical complications, both microvascular and macrovascular complications, in addition to other secondary complications (Stirban et al., 2014; Quehenberger et al., 2000).

Natural products have been proven to be relatively safe for humans. They are nontoxic, inexpensive and have less or no side effects (Konda et al., 2019). Consumption of some plant extracts can prevent the formation of AGEs. Extracts or natural compounds possess antioxidant and antiglycation capabilities which could provide potential therapeutic candidates to treat diabetes and prevent diabetes complications (Tripathi and Srivastava, 2006). Seaweed, i.e., algae is one example of an excellent and abundant source of exogenous antioxidants and antiglycation components. Marine macroalgae and their substances have antidiabetic abilities and thus could be used for diabetic treatment (Samaddar and Koneri, 2019). *Kappaphycus alvarezii* (Doty) Doty ex P.C.Silva is one of the largest tropical macroalgae with relatively high growth rates compared to the other types of seaweed (Ramdhan, 2018).

In Indonesia, seaweed cultivation generally uses the *Eucheuma* genus (Soenardjo, 2011). These algae contain various compounds, such as dietary fiber, vitamin C, α-tocopherol, minerals, fatty acids, and protein. *Kappaphycus alvarezii* (Doty) Doty ex P.C.Silva is rich with antioxidants and can significantly prevent tissue damage by stimulating the wound healing process. It also acts as an anti-inflammatory agent due to their phenolic compounds and derivatives, including simple phenols, flavonoids, hydroquinone, triterpenoids, phenyl proponoids, tannins, lignins, and many other substances. These contain aromatic rings and hydroxyl groups, which will determine the destructive power of radical compounds (Selvan et al., 2014; Chojnacka et al., 2012).

The use of crude extracts as medicinal plants involves several difficulties, i.e. the amount and type of bioactive compounds can vary according to the location and collection season. The bioactive molecules could also become strong poisons when they are overconsumed. On the other hand, when they are consumed in a lower amount, the bioactive compounds could be ineffective due to suboptimal doses, and/or the quick loss of drug properties during the storage process. Therefore, it is important to isolate and identify bioactive molecules from a plant extract. Bioassay-guided fractionation from plant extracts based on their biological activity, in combination with chromatographic separation technique, could be used to isolate these active molecules (Malviya and Malviya, 2017).

The use of animal models in diabetes mellitus research is very important to understand various aspects of pathogenesis and to find new therapeutic methods. Animal diabetic models are useful in biomedical studies because they can provide new insights into human diabetic research. In this regard, rodents are used as the most common animal models due to their small size, short generation intervals, easy availability and economic considerations (Singh and Pathak, 2015). Hyperglycemic conditions are usually induced in those rats using Streptozotocin (STZ) and nicotinamide (NA). In experimental animals, the most prominent diabetogenic chemical that is often used is STZ among other substances that also have a diabetes effect (alloxan, vactor, dithizone, and 8-hydroxyquinolone). Alloxan destroys pancreatic beta cells through oxidative stress and, compared to STZ, has lower effectiveness and side effects, including liver and kidney damage. The type 2 diabetes-induced rat model with STZ is based on the protective effect of NA against the β-cytotoxic effects of STZ (Ghasemi et al., 2014). Administration of 230 mg/kg intraperitoneal NA 15 min before 65 mg/kg intravenous STZ resulted in an increase glucose concentration of 5–11 mmol/l (90–198 mg/dL) (Masiello et al., 1998).

This study was conducted to explore the ability of the active fraction of *Kappaphycus alvarezii* (Doty) Doty ex P.C.Silva, in inhibiting the glycation process and subsequently to examine the effect of that active fraction on fasting plasma glucose, glycated albumin, and CML levels and renal RAGE gene expression.

## 2 Results

**Figure 1:**
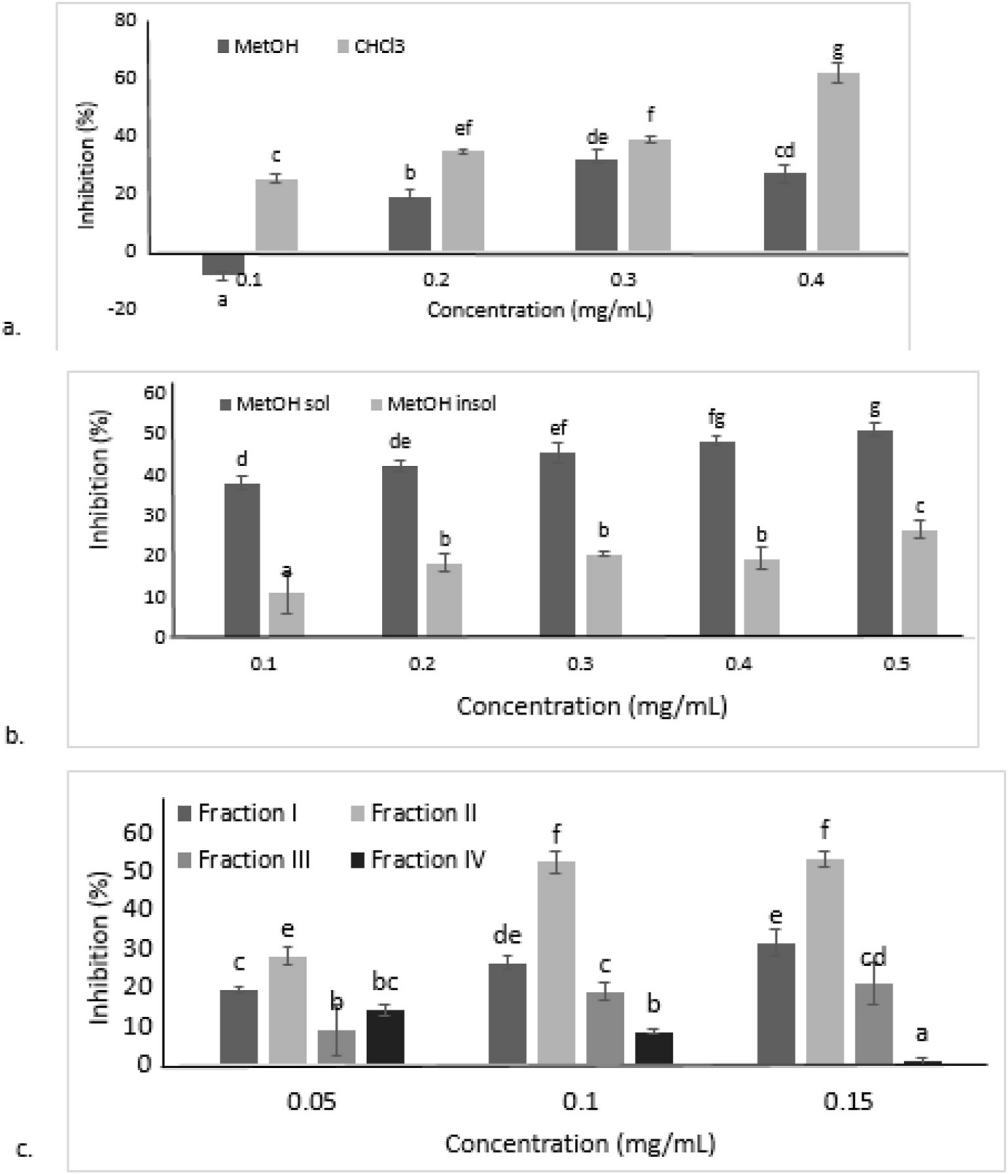
Percentage of inhibition of BSA-Glucose test results using Bioassay Guided Fractionation. a. Methanol and chloroform extracts. b. Methanol soluble and insoluble extract. c. Fractionation extracts in the formation of AGEs. Each value represents the mean ± SD after incubation for 6 days at 37 °C. Different letters show significant differences (*p* < 0.05).

Chloroform and methanol extract glycation tests showed a significant difference (*p* < 0.05). The percentage of inhibition of glycation process in chloroform extract was higher than it was in methanol extract (Figure 1a). Chloroform extract with a concentration of 0.4 mg/mL showed the highest percentage of inhibition among other treatments (62.4 ± 3.45%). In addition, the IC_50_ value of chloroform extract was lower (0.33 ± 0.01 mg/mL) when compared to the IC_50_ value of methanol extract (0.52 ± 0.03 mg/mL). Chloroform extract had higher percentage of inhibition therefore, it was then used for the partition step with methanol.

A significant difference between the treatments using methanol-soluble and methanol-insoluble extracts was also observed (*p* < 0.05). The highest percentage of inhibition was produced by treatment using extracts that were soluble in methanol at the concentration of 0.5 mg/mL (51.10 ± 1.64%) as seen in Figure 1b. IC_50_ values obtained from the two treatment groups showed that the methanol-soluble extract had lower IC_50_ (0.45 ± 0.05 mg/mL) compared to the methanol-insoluble extract (1.25 ± 0.05 mg/mL).

Antiglycation tests from all obtained fractions (Fraction I-IV) demonstrated a significant difference among different treatments with fractions from red algae (*p* < 0.05) as shown in Figure 1c. Fraction II treatment at a concentration of 0.15 mg/mL had the highest percentage of inhibition (53.37 ± 1.92%) and the lowest IC_50_ value (0.12 ± 0.01 mg/mL).

**Figure 2:**
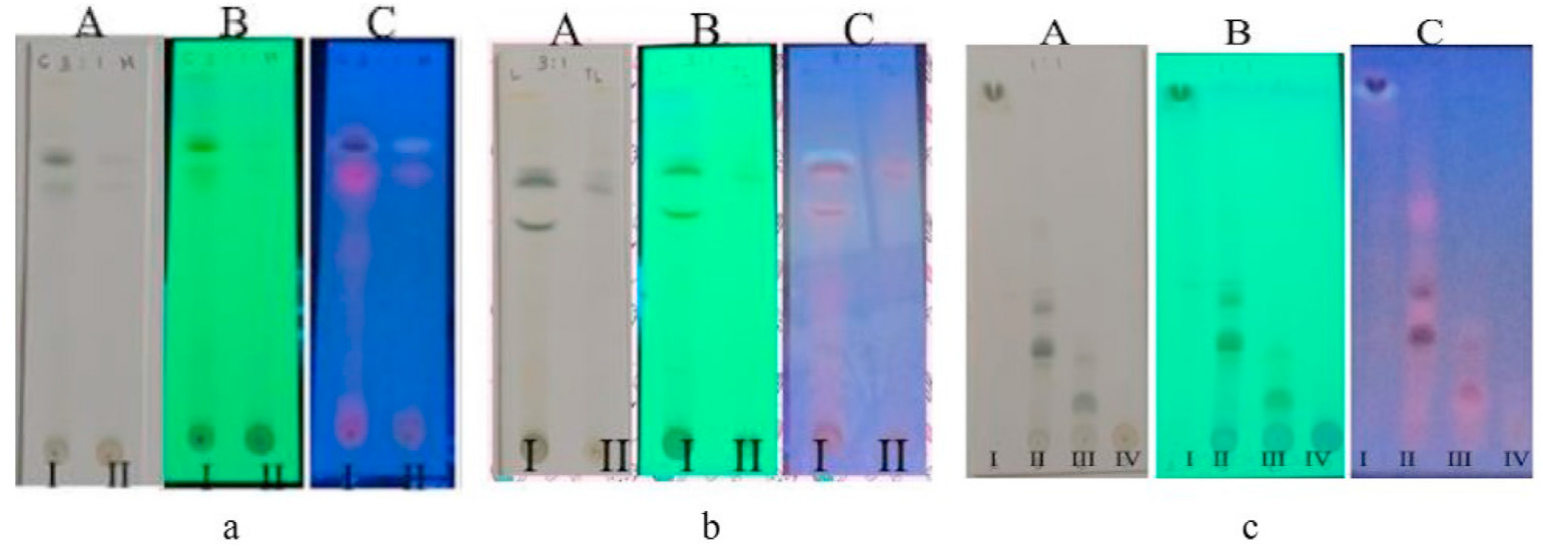
TLC profile of all extracts. a. Methanol extract (I) and chloroform extracts (II). b. methanol-soluble extracts (I) and methanol-insoluble extracts (II). c. Fraction I (I), Fraction II (II), Fraction III (III) and. Fraction IV (IV). A = visible light, B = 254 nm UV light, C = 365 nm UV light. Stationary phase = silica gel 60 F254; Mobile phase = wash benzene: ethyl acetate (3: 1 v/v).

The separation between polar and non-polar compounds of the extraction process was confirmed using Thin Layer Chromatography (TLC) (Figure 2a). The TLC results showed that the compounds of chloroform and methanol extracts were completely separated with the difference spots between the two groups. The partitioning of chloroform extract and the fractionation of methanol-soluble extracts were confirmed with TLC, as shown in Figure 2b and c, respectively. The spot differences or the absence of the same spots between the extracts indicated the success of the partition and fractionation process. Furthermore, all IC_50_ values treated using Bioassay-guided Fractionation (BSA-Glucose method) are shown in Table 1.

**Table 1:** IC_50_ values for the antiglycation test of all extracts using Bioassay-guided Fractionation.

Extracts	IC_50_ (mg/mL)
MeOH	0.52 ± 0.03^b^
CHCl3	0.33 ± 0.01^b^
MeOH soluble	0.45 ± 0.05^b^
MeOH insoluble	1.25 ± 0.05^c^
Fraction I	0.31 ± 0.08^b^
Fraction II	0.12 ± 0.01^ab^
Fraction III	0.65 ± 0.60^b^
Fraction IV	-0.22 ± 0.02^a^

Each value represents the mean ± SD. Different letters indicate significant differences (*p* < 0.05).

IC_50_ results of all extracts showed a significant difference between treatments (*p* < 0.05). The data in Table 1 show that fraction II had the lowest IC_50_ compared to the other extracts. In this study, fraction II had a higher inhibitory activity because fractions contained more active compounds than the extract with the same concentration. The kind of active compound contained in the active fraction was also not as much as that contained in the extract. The difference in polarity of the solvent for extraction and fractionation could also affect the inhibitory activity.

### 2.1 *In vivo* test results

The selected active fraction obtained from the bioassay-guided fractionation was then used for the *in vivo* test using hyperglycemia-induced rats (5 rats per group). At the end of the study, rats in each group were in good health with an average weight in each group between 207.6-279.8 g. All rats in this study were further analyzed.

#### 2.1.1 Plasma glucose levels

**Figure 3:**
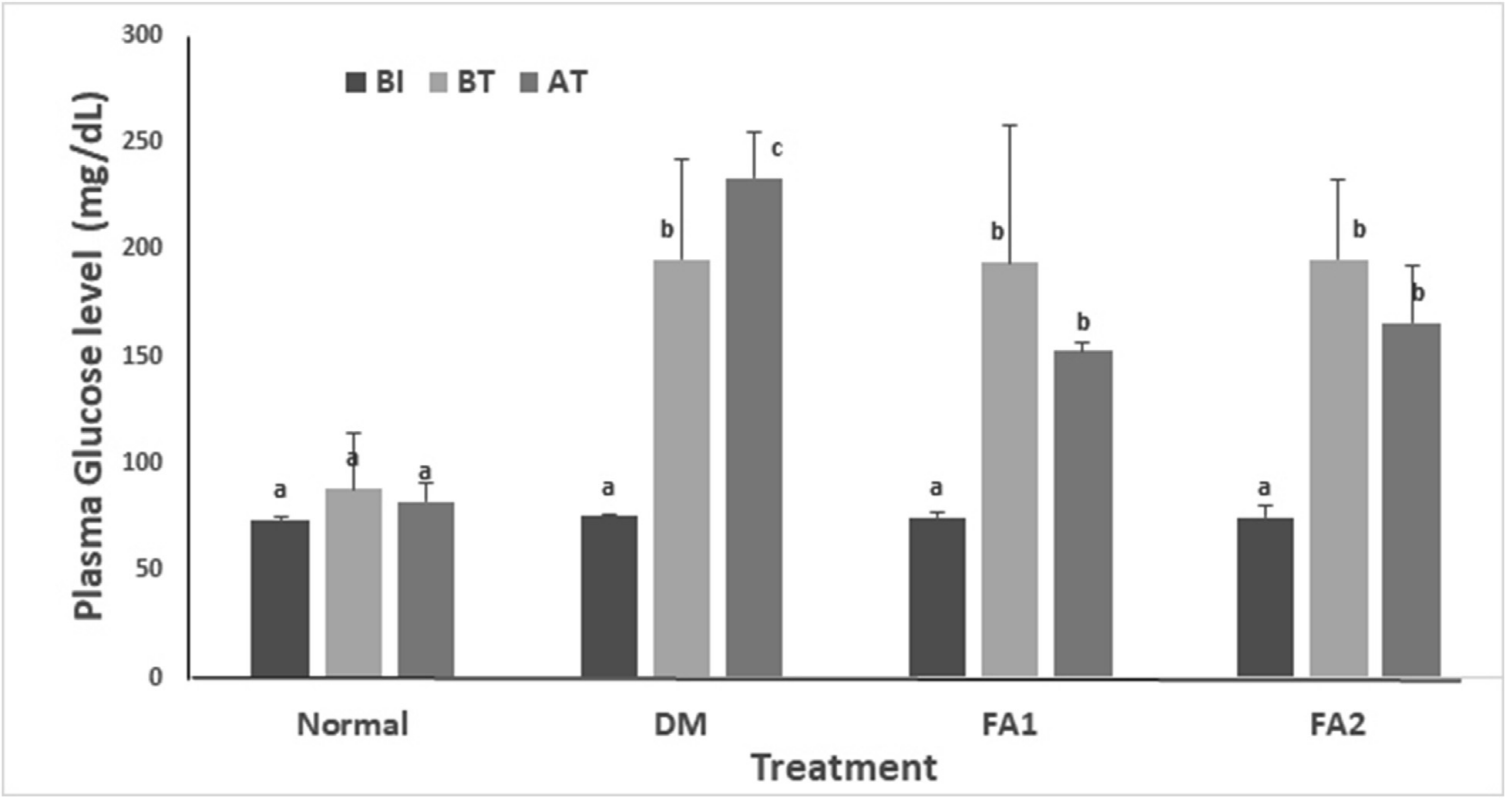
Plasma glucose levels. Normal = non-diabetic rat group. DM = diabetic rat group. FA1 = diabetic rat group treated with 0.17 mg/mL active fraction. FA2 = diabetic rat group treated with 0.255 mg/mL active fraction. BI = before diabetic induction using STZ and NA. BT = before treatment. AT = after treatment. Each value represents the mean ± SD of each treatment group. Different letters show significant differences (*p* < 0.05).

Plasma glucose levels measured in the diabetic rat group without active fraction treatment was the highest than other diabetic rat groups (233.60 ± 21.29 mg/dL) while the lowest was found in the nondiabetic rat group (82 ± 9.06 mg/dL). There was a significantly different plasma glucose level before and after hyperglycemia induction with STZ and NA (paired T-Test, *p* < 0.05). The STZ-NA induction had successfully induced the increase of plasma glucose level up to more than 61%. Treatment with active fraction of red algae on the diabetic rat group did not provide significant results for the decrease of glucose levels (*p* > 0.05). Nevertheless, treatments with concentrations of 0.17 mg/mL and of 0.255 mg/mL active fractions could reduce glucose levels as much as 21.13% and 15.34%, respectively (Figure 3).

#### 2.1.2 Plasma glycated albumin (GA) levels

**Figure 4:**
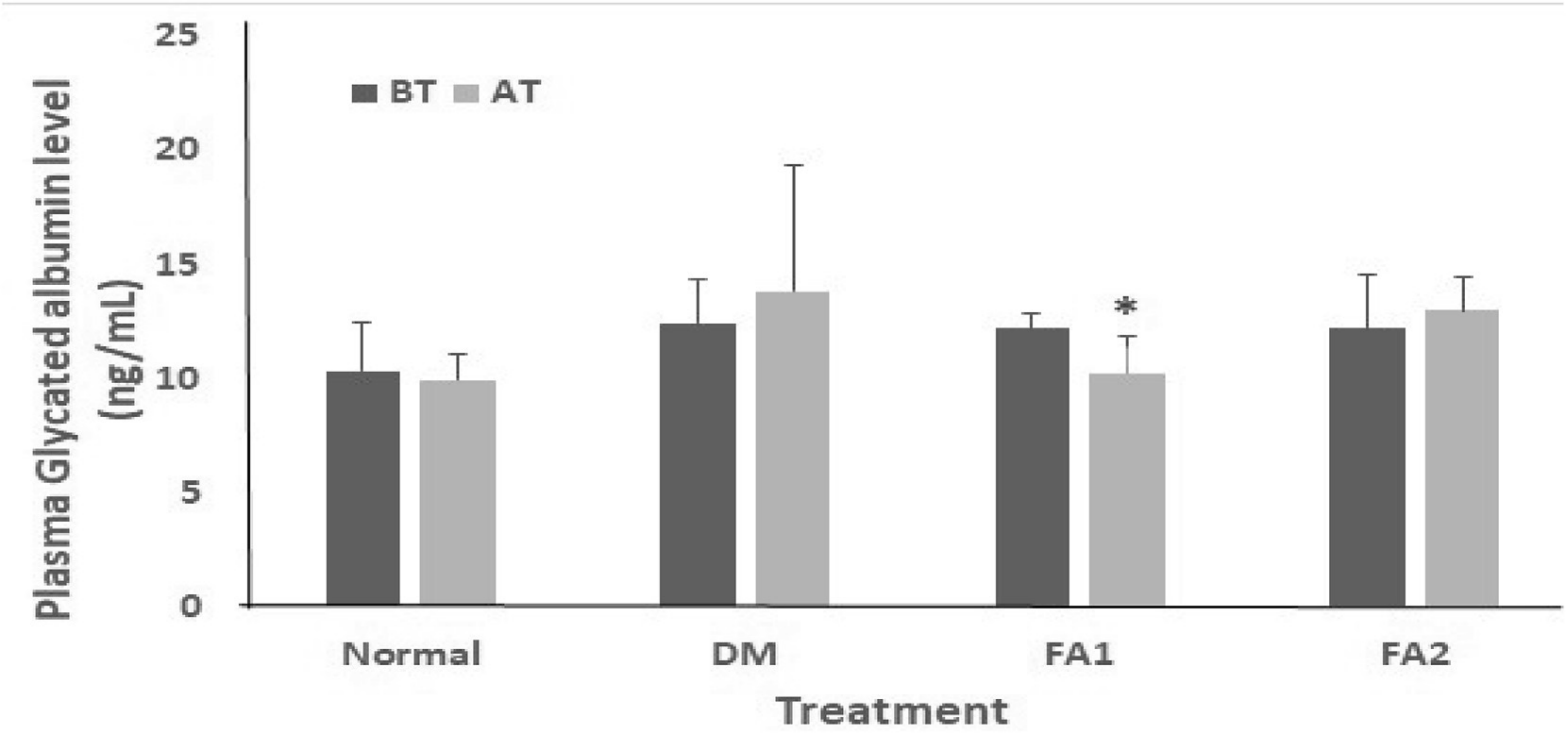
Plasma GA levels. Normal = non-diabetic rat group. DM = diabetic rat group. FA1 = diabetic rat group treated with 0.17 mg/mL active fraction. FA2 = diabetic rat group treated with 0.255 mg/mL active fraction. BT = before treatment. AT = after treatment. Each value represents the mean ± SD of each treatment group. ∗ show significant differences (*p* < 0.05).

Plasma GA levels only showed significant difference in the group of diabetic rats with 0.17 mg/mL active fraction treatment (paired T-Test, *p* < 0.05). The diabetic rat group without treatment showed the highest plasma GA levels (13.85 ± 5.49 ng/mL) with an increase of 10.47%. In the group with treatment of 0.17 mg/mL active fraction, plasma GA levels decreased significantly as much as 15.75% (10.27 ± 1.62 ng/mL), whereas treatment with active fraction of 0.255 mg/mL did not significantly increase the plasma GA levels with a result of only 6% (12.99 ± 1.43 ng/mL) (Figure 4).

#### 2.1.3 Plasma Nε- (carboxymethyl) lysine (CML) levels

**Figure 5:**
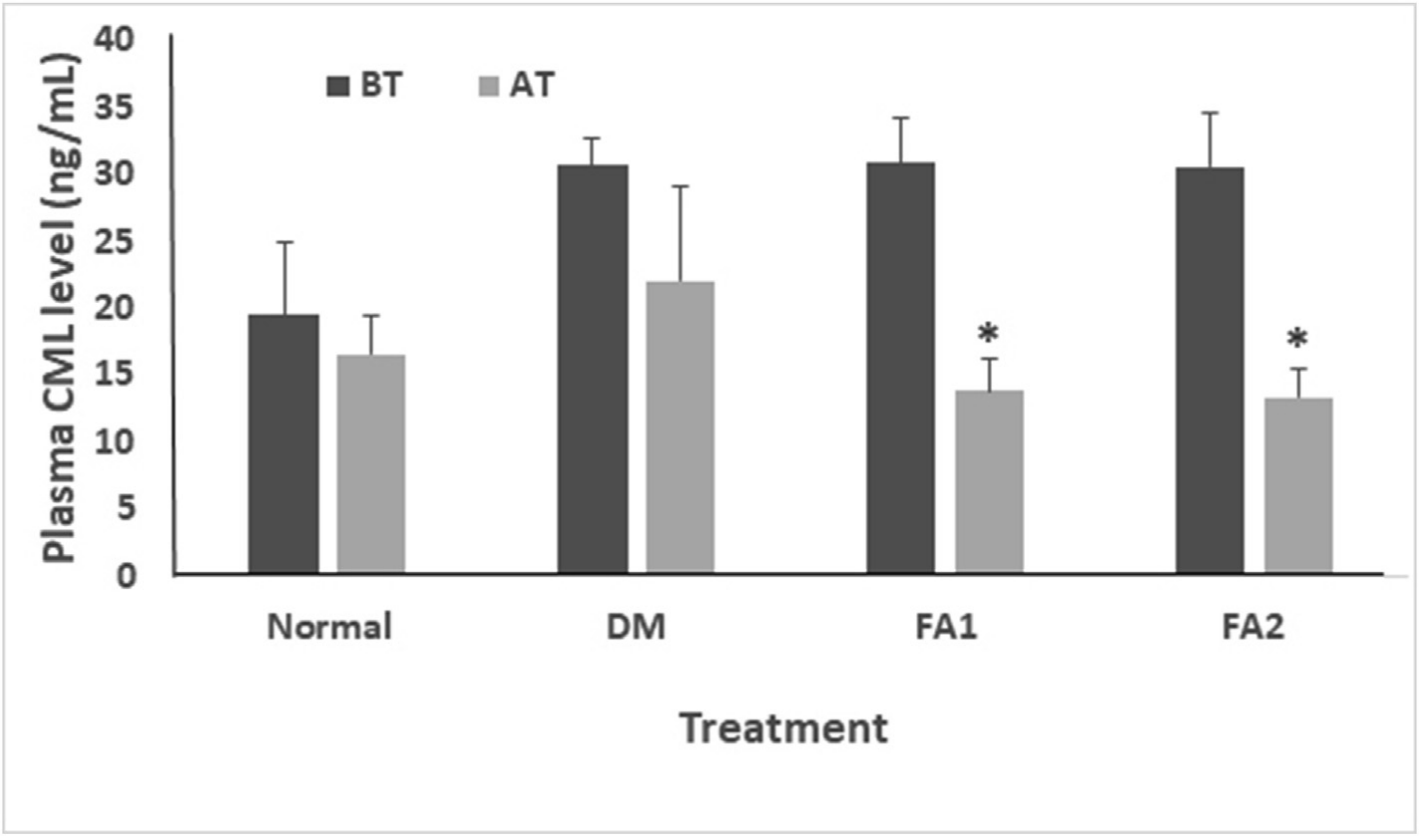
Plasma CML levels. Normal = non-diabetic rat group. DM = diabetic rat group. FA1 = diabetic rat group treated with 0.17 mg/mL active fraction. FA2 = diabetic rat group treated with 0.255 mg/mL active fraction. BT = before treatment. AT = after treatment. Each value represents the mean ± SD of each treatment group. ∗ show significant differences (*p* < 0.05).

Figure 5 shows that plasma CML levels in both treatments at the concentrations of 0.17 and 0.255 mg/mL showed a significant decrease of 55.13% (13.86 ± 2.30 ng/mL) and 56.21% (13.39 ± 2.05 ng/mL), respectively (*p* < 0.05).

#### 2.1.4 Renal RAGE gene expression analysis

**Figure 6:**
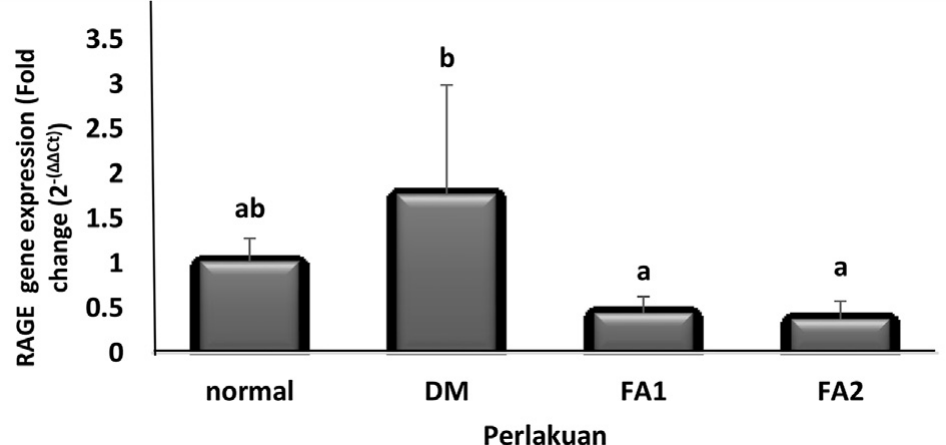
Renal RAGE gene expression. Normal = non-diabetic rat group. DM = diabetic rat group. FA1 = diabetic rat group treated with 0.17 mg/mL active fraction. FA2 = diabetic rat group treated with 0.255 mg/mL active fraction. Different letters show significant differences (*p* < 0.05).

Renal RAGE gene expression analysis showed a decrease in RAGE gene expression, both in the diabetic rat groups treated with active fractions of 0.17 mg/mL (0.45 times) and of 0.255 mg/mL (0.38 times) (Figure 6), compared to the nondiabetic rat group. Results showed that the hyperglycemic condition can increase the RAGE gene expression in the kidneys. Moreover, there was an increase in renal RAGE gene expression of the diabetic rat group that was not treated with red algae active fraction by 1.77 times, compared to the non-diabetic rat group.

#### 2.1.5 High Resolution Mass Spectrometry (HRMS) analysis

HRMS could identify 707 compounds from the active fractions obtained from bioassay guided fractionation, some of which have been known to reduce glucose levels in the blood and demonstrated anti-glycation activity (Table 2).

**Table 2:** Results of analysis of active fraction content from red algae with antiglycation ability using HRMS.

No	Name	Formula	Molecular Weight	RT [min]	Area (Max.)	% relative	mzCloud Best Match	mzCloud Best Sim. Match	MS2
1.	Pheophorbide A	C_35_H_36_N_4_O_5_	592.267	29.898	273,975,531.7	7.27			ddMS2 for preferred ion
2.	Cafestol	C_20_H_28_O_3_	316.203	22.996	13,247,102.47	0.35	82.4		ddMS2 for preferred ion
3.	Shogaol	C_17_H_24_O_3_	276.172	21.776	5,197,299.59	0.14	83.8		ddMS2 for preferred ion
4.	Thymol	C_10_H_14_O	150.104	3.375	4,872,277.27	0.13		92.8	ddMS2 for other ion
5.	Cinnamic acid	C_9_H_8_O_2_	148.052	3.402	4,936,572.72	0.13		82.7	ddMS2 for preferred ion
6.	Kahweol	C_20_H_26_O_3_	314.188	24.869	2,482,362.849	0.06	85.3		ddMS2 for preferred ion
7.	p-cymene	C_10_H_14_	134.109	3.393	1,280,953.71	0.03		86.1	ddMS2 for other ion
8.	Pyrogallol	C_6_H_6_O_3_	126.032	3.311	702,966.266	0.02		67.3	ddMS2 for preferred.ion
9.	(E)-p-coumaric acid	C_9_H_8_O_3_	164.047	3.328	803,138.4894	0.02		85.3	ddMS2 for preferred ion
10.	(-)-Lupinine	C_10_H_19_NO	169.146	22.893	249,069.529	0.01			No MS2
11.	(E)-Ferulic acid	C_10_H_10_O_4_	194.058	17.175	362,404.05	0.01		82	ddMS2 for preferred ion
12.	Putrescine	C_4_H_12_N_2_	88.1005	3.845	463,575.4833	0.01	60.5		ddMS2 for preferred ion
13.	Anacardic acid	C_22_H_36_O_3_	348.266	24.644	177,735.4029	<0.01			No MS2

## 3 Discussion

Bioassay-guided fractionation is widely used to isolate active ingredients from plants based on different physicochemical properties. It consists of several extraction steps, starting from assessing biological activity, followed by subsequent separation and testing processes. Chromatography is then used to fractionate the extract, finding the active fraction. Each extraction step is evaluated in the bioassay system as such only the active extract is further separated. In this way, the inhibitory activity will be higher and IC_50_ value will be lower.

The bioassay-guided fractionation process began by separating polar compounds using methanol- and separating non-polar compounds using chloroform-solvents. The two extracts were then monitored by the bioassay (BSA-Glucose) method and the chromatography pattern was also observed. In the BSA-Glucose method, the glycation inhibition process can be seen from the formation of fluorescent glycated protein (Muramatsu et al., 2019). This bioassay-guided fractionation method has been previously applied to detect the presence of active fractions from *T. diversifolia* (Hemsley) A. Gray, which have antiplasmodial potential (Syarif et al., 2018). Moreover, it has been widely used in the initial isolation stages of compounds from natural materials, such as the tagitinin C compound from *T. diversifolia* (Wahyuningsih et al., 2015) and zerumbone from *Zingiber zerumbet* (Murini et al., 2018).

Treatment of diabetic rats with active fractions of red algae at the concentrations of 0.17 and 0.255 mg/mL could reduce glucose levels as much as 21.13%, and 15.34%, respectively, although the plasma glucose reduction was not significant. This result could be explained by the very small concentration of the active substances for reducing glucose in the active fraction, therefore, the decrease of glucose plasma level was not significant. Nevertheless, the decrease of plasma glucose level was due to several chemical compounds contained in the active fraction that are proven to reduce blood glucose levels. Some potential antidiabetic compounds identified in the active fractions of red algae in this study are shown in Table 2.

Cafestol and kahweol are natural diterpenes. Previous research showed a decrease of blood glucose levels in the group of mice treated with kahweol (Baek et al., 2017). Kahweol induces AMP-activated protein kinase (AMPK) activation. AMPK regulates glucose metabolism by increasing glycolysis, activating 6-phosphofructo-2-kinase/fructose-2,6-bisphosphatase, and suppressing glycogen synthesis through inhibition of glycogen synthase. AMPK increases glucose absorption by increasing the expression of glucose transporter 4 and hexokinase II in skeletal muscle cells (Ren et al., 2019). Cafestol also showed antidiabetic ability. Cafestol intervention significantly reduces blood fasting glucose and glucagon. It also increases insulin secretion and sensitivity in KKAy mice (Tedong et al., 2010). Other compounds with the same antidiabetic activity include anacardic acid (Frimat et al., 2017) and cinnamic acid that are known to be effective in reducing blood glucose levels in animal models (Adisakwattana et al., 2012).

Protein glycation is a complex cascade of steps, starting with the non-catalytic binding of sugar, such as glucose, fructose or its derivatives, to the amino groups of a protein. A rearrangement of functional protein molecules occurs before the glycated protein is produced, followed by the formation of crosslinking of these proteins. In diabetic patients, the formation of AGEs is increased, resulting in damaging effects on vital tissues such as the retina, neurons, nephrons, and the heart (Shah et al., 2013).

Different therapeutic approaches have been developed to limit the adverse effects of AGEs. First, reducing the rate of absorption of AGEs through an adjusted diet or by reducing the absorption of the AGEs in the gastrointestinal tract. Second, the presence of inhibitors could stop the formation of AGEs in the early stages. Third, administering several active molecules can damage the formed AGEs. Finally, interaction of AGEs with its receptor, RAGE, is interfered with either by using several traps or antibodies, thereby avoiding the intracellular cascade and its effects (Liu et al., 2018).

**Figure 7:**
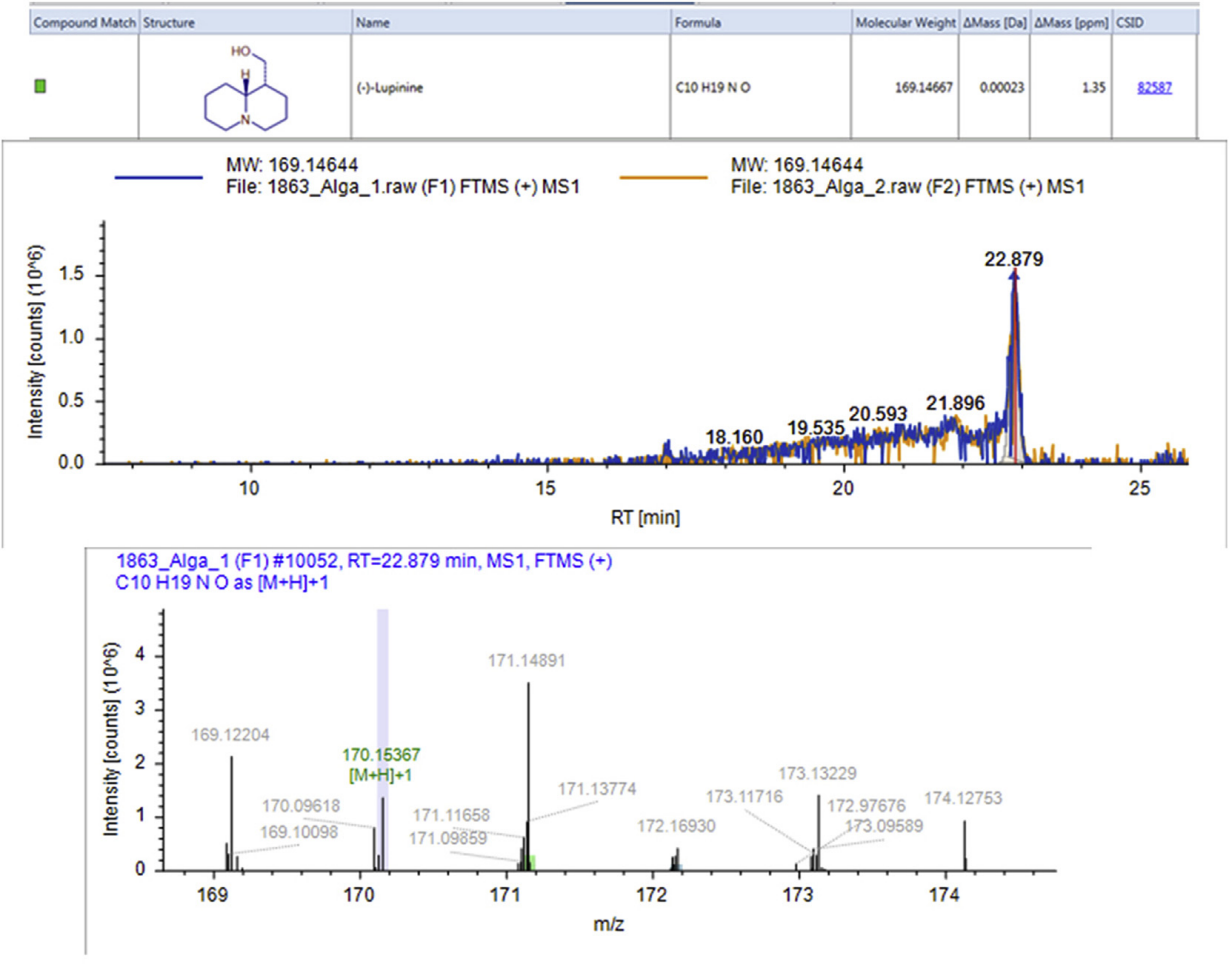
HRMS chromatogram of (-)-Lupinine

*In vitro* test with the BSA-Glucose method showed the ability of active fraction II from red algae to inhibit the glycation process at a concentration of 0.15 mg/mL with IC_50_ of 0.12 ± 0.01 mg/mL. Some of the compounds in the active fraction of red algae have antiglycation abilities, for example, a phenolic compound, ferulic acid. The antiglycation effect of this compound was investigated using the BSA-Glucose method. Ferulic acid has electron donating groups in the benzene ring (3-methoxy and 4-hydroxyl) which can form phenoxy radicals. Ferulic acid is inhibited in the formation of AGEs because of its ability in capturing free radicals, metal chelating, and trapping carbonyl. Ferulic acid prevents the formation of AGEs by acting as an antioxidant, binding to amino groups, inhibiting sugar autoxidation and early degradation of Maillard Reaction Products (MRP) (Yeh et al., 2017; Silván et al., 2011). Another compound that works by binding to amino groups in BSA are thymol, and p-Cymene (Abbasi et al., 2018). Lupinine, an alkaloid compound (Figure 7), interacts by reducing sugars, so that these sugars cannot bind to the amino groups of a protein (Abbas et al., 2016).

In the *in vivo* test, GA levels significantly decreased in the diabetic rat group with 0.17 mg/mL active fraction, but not with the administration of the 0.255 mg/mL active fraction. In this study, the lower active fraction dosage was more optimal in inhibiting GA formation. It can be due to the higher concentration of active compounds, enabling interaction between these active compounds, so that it can reduce the interaction of active compounds with target compounds such as glucose and albumin (D'Archivio et al., 2010). The active fraction used in this study is a collection of several compounds. This could also be seen in the TLC profile, where some spots were still formed. The HRMS test also showed the presence of several compounds. However, it is not certain which compounds act in preventing the formation of GA. To find out more clearly about the compounds that work in inhibiting the formation of GA, further research is needed to isolate the active compounds using the same bioassay.

The CML levels dropped significantly in both active fraction treatment groups. Formation of CML can be inhibited through antioxidant activity to reduce the formation of AGEs by preventing the oxidation of Amadori products and metal-catalyzed glycoxidation (Dil et al., 2019). Inhibition of AGEs formation through methylglyoxal (MGO) blockage has been demonstrated in studies using shogaol (Nonaka et al., 2019) and putrescine (Park et al., 2017). Previous research showed the ability of shogaol to reduce the formation of CML in a BSA/Fructose solution (Malakul and Pengnet, 2017). The antioxidant properties of shogaol prevented fructose and Amadori products from oxidation, leading to the inhibition of AGEs production. The same result was found in a recent study using pyrogallol (Zhang et al., 2019) and p-Coumaric acid (Moselhy et al., 2018) which showed their antiglycation ability by inhibiting further oxidation of glycated proteins and oxidation of metal-catalyzed glucose which leads to the formation of AGEs. Protein glycation activity can also be inhibited by cinnamic acid (Adisakwattana et al., 2012) using the BSA-fructose method. Cinnamic acid and its derivatives reduce fructosamine levels, CML formation, and cross-β amyloid levels. Non-enzymatic glycation of proteins has been reported to stimulate protein aggregation and amyloid deposition (Iannuzzi et al., 2014). Protein glycation accelerates the formation of protein aggregation and amyloid cross β-structures leading to altered protein structure, stability, and function (Adisakwattana, 2017). Islet amyloid deposits may play an important role in the loss of β cells and the progressive decline in insulin secretion characteristic of type 2 diabetes (Marzban et al., 2003).

It was previously known that hyperglycemic conditions can increase RAGE expression (Yao and Brownlee, 2010). The RAGE gene expression obtained in this study showed enhancement by 1.77 times in the group of diabetic rats that were not treated with active fraction compared the non-diabetic control group. Decrease in CML, in this study, was followed by a decrease in the expression of the RAGE gene. CML is one of the major AGE structures found in tissue and blood plasma, and is a ligand of RAGE (Xue et al., 2012; Schmidt et al., 2000). Treatments with active fractions of red algae at concentrations of 0.17 mg/mL and 0.255 mg/mL showed a statistically significant decrease of renal RAGE gene expression 0.45 times and 0.38 times, respectively. Previous research showed that RAGE was expressed in normal podocytes and its concentration was increased in diabetic nephropathy. CML is the main AGE found in the renal basal membrane of diabetic nephropathy, and its accumulation involves an increase of RAGE regulation in podocytes (Yuan et al., 2017; Tanji et al., 2000).

**Figure 8:**
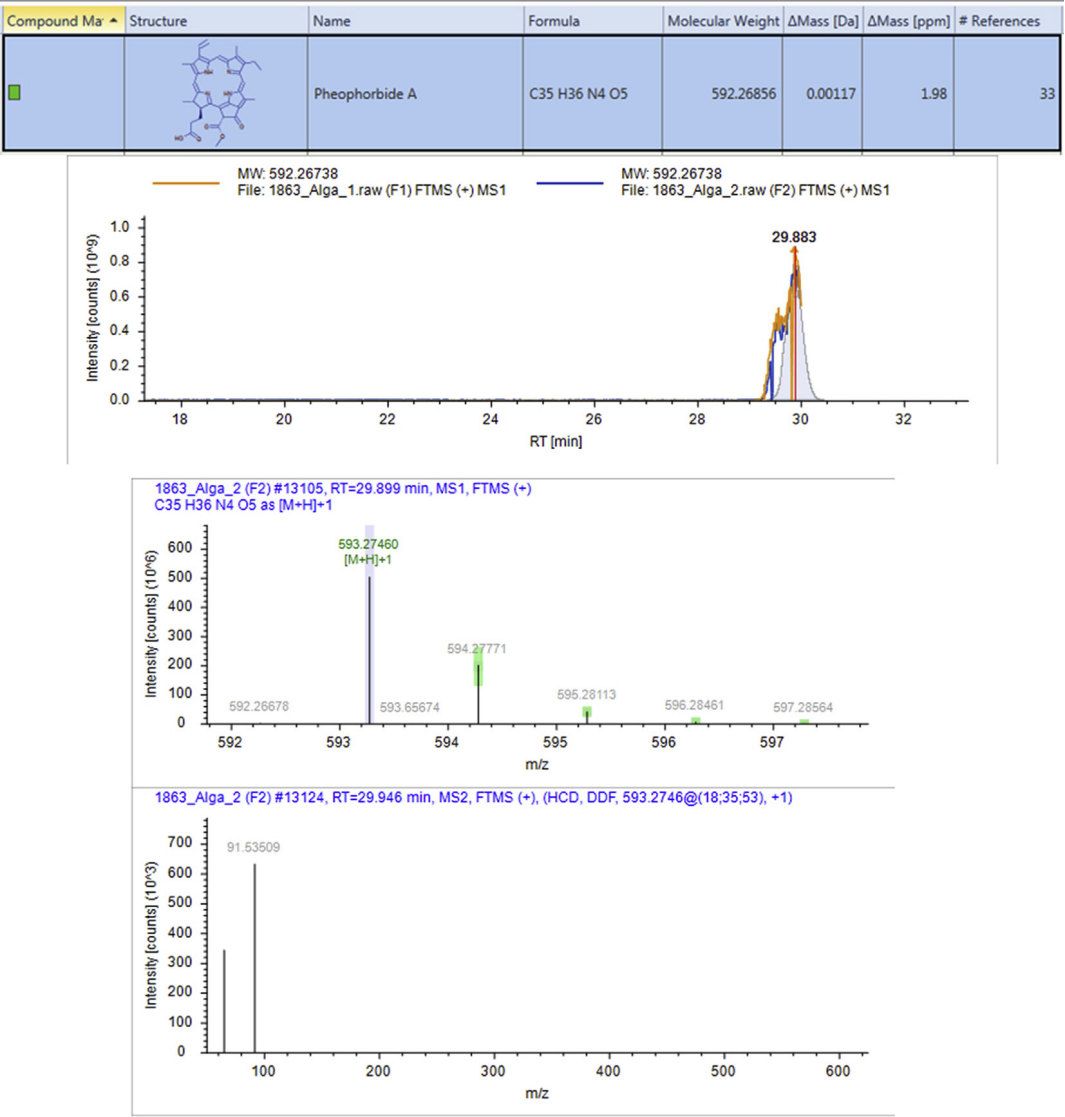
HRMS chromatogram of Pheophorbide A

Pheophorbide A is the most common compound found in the active fractions of algae (7.27%) in this study (Figure 8). The previous study showed that this compound has significant inhibitory activity on RAGE mRNA expression and inflammatory cytokines induced by the presence of AGEs (Hong et al., 2015). Pheophorbide A had a protective effect and prevented diabetes complications by inhibiting the formation of AGEs.

## 4 Conclusions

In conclusion, fraction II is the main anti-glycation agent of *Kappaphycus alvarezii* (Doty) Doty ex P.C.Silva with the lowest IC_50_ value (0.12 ± 0.01 mg/mL) and a high percentage of inhibition (53.37 ± 1.92%). The administration of this active fraction in diabetic rats could reduce plasma GA level of 15.75% with concentration of 0.17 mg/mL and plasma CML levels of 55.13% and 56.21% at concentrations of 0.17 and 0.255 mg/mL, respectively, although the reduction of plasma glucose levels was not significant. In addition, RAGE gene expression in the diabetic group of rats treated with active fraction from red algae was also lower than in the diabetic group without treatment.

## 5 Materials and methods

### 5.1 Plant material

*Kappaphycus alvarezii* (Doty) Doty ex P.C.Silva were obtained from Lombok Tengah, Nusa Tenggara Barat, Indonesia in February 2019. The algae were identified at Biology Laboratory, Faculty of Mathematics and Science, Universitas Mataram, Mataram, NTB, Indonesia with identification certificate number 03/UN18.7/LB/2019.

### 5.2 Animals

Twenty Wistar male rats used in this study were from Bogor Life Science and Technology (BLST) Company, Bogor, Indonesia. All animal experiment procedures were approved by the Medical and Health Research Ethics Committee of the Faculty of Medicine, Public Health and Nursing, Universitas Gadjah Mada, Yogyakarta, Indonesia with Ref. No.: KE/FK/0564/EC/2019.

### 5.3 Instruments and chemicals

The instruments used were oven (Memmert, Germany), centrifuge (Hitachi 18PR/5, Automatic high-speed refrigerated centrifuge), UV lamp, spectrofluorometer (Shimadzu RF 6000), High Resolution Mass Spectrometry (HRMS) using Thermo Scientific™ Dionex™ Ultimate 3000 RSLCnano UHPLC coupled with Thermo Scientific™ Q Exactive™ High Resolution Mass Spectrometer. The chemicals used were purchased from Merck, Inc. The chemicals were methanol, chloroform, ethyl acetate, ether, silica gel GF254, BSA, sodium azide, glucose, silica plate, wash benzene, phosphate-buffered saline (PBS), STZ and NA, glucose GOD-PAP Biolab Reagents, ELISA kit for glycated albumin, Nε-(carboxymethyl) lysine (CML) (Bioassay Technology Laboratory), RNA later solution (invitrogen), RNAprep Pure Kit (Invitrogen), iScript™cDNA Synthesis Kit Cat. No. 1708890, Sso Fast ™ EvaGreen ® Supermix Cat No 172–5200, Eppendorf tube 1.5 mL, qPCR tube 200 μL (Bio-Rad), tip, RAGE primer, β-actin primer.

### 5.4 Study design

In this study, the sample size calculation was based on the resource equation, which minimum sample is *n* = 10/*k* + 1 and for maximum sample is *n* = 20/*k* + 1 with *k* = number of groups, and *n* = number of subjects per group (Arifin and Zahiruddin, 2017), because this study was designated as 4 groups, the minimum sample was 10/4 + 1 = 3.5 and the maximum sample was 20/4 + 1 = 6. Therefore, the sample size used in this study was 5 Wistar male rats in each group. Twenty male Wistar rats, with an average weight of 200 g, aged 8 weeks were acclimated for 1 week in the laboratory. Each rat was placed in an individual cage that had been cleaned. Room temperature ranges from 25-28 °C with a 12-hour lighting circulation. Rats were given standard feed and drink *ad libitum*.

### 5.5 Extraction, partition, and fractionation

The thalli of red algae (*Kappaphycus alvarezii* (Doty) Doty ex P.C.Silva) were cut into pieces, sun dried, and powdered. A thousand grams of dry powder were extracted by maceration using chloroform and the residue was extracted with methanol. The extracts were identified by thin layer chromatography (TLC). The antiglycation activity was observed with BSA-glucose method. Twenty eight grams of active extract (chloroform) was partitioned using methanol, to get methanol-soluble and methanol-insoluble compounds. They were identified using TLC and tested for its antiglycation activity. The active extract (methanol soluble, 3 g) was then fractionated by liquid chromatography that was modified using vacuum with a stationary phase of silica gel GF254. The mobile phase used was wash benzene (100%), wash benzene: ethyl acetate (9:1; 8:2; 7:3; 6:4; 5:5; 4:6; 3:7; 2:8; 1:9 v/v) ethyl acetate (100%), and chloroform: methanol (1:1 v/v). TLC identification of liquid chromatography yielded 12 fractions that were combined into 4 fractions (Fraction I-IV) based on spot similarity in TLC, then used for antiglycation test. The active fraction that had the highest percentage of inhibition and lowest IC_50_ value from BSA-glucose test was fraction II, so it was used for *in vivo* study.

### 5.6 Compound analysis with High Resolution Mass Spectrometer (HRMS)

To find out the compounds contained in the active fraction, an analysis was performed using High Resolution Mass Spectrometer (HRMS) (Thermo Scientific ™ Dionex ™ UltiMate 3000 RSLC nanoplan UHPLC coupled with Thermo Scientific Q Exactive ™ High Resolution Mass Spectrometer). The mobile phase used was A = Water +0.1% Formic Acid and B = Acetonitrile +0.1% Formic Acid. The separation was conducted under the following gradient: 0 min 5% B; 0–2 min 5% B; 2–15 min 60% B; 15–22 min 95% B; 22–25 min 95% B; 25 min 5% B; 25–30 min 5% B. Analytical column used was Hypersil GOLD aQ 50 mm × 1 mm x 1.9 μm with flow of 10 μL/min, injection volume of 5 μL, the total time of the chromatographic was 30-minute run time. The resolution used were 70,000 FWHM for MS1 and data-dependent MS2 at 17,500 FWHM. This study used Heated Electrospray Ionization (H-ESI) in positive mode. Spray Voltage used in this study was 3.8 kV. The flow rate for Sheath gas was 15 and Aux gas was 7. The capillary temperature was 250 °C. The mass range used was between 50 - 750 m/z. Acquired data were profile data. Compound identification was done using Thermo Scientific ™ Compound Discoverer Software.

### 5.7 *In vitro* glycation of Bovine serum albumin (BSA)

BSA was incubated with glucose in PBS (20 mM, pH 7.4) and extract containing 0.02% sodium azide at 37*◦*C with a final concentration of BSA (5 mg/mL), glucose (200 mM), and samples (0.1–0.5 mg/mL). The solution was incubated in the dark at 37 °C for 6 days. The AGE formation was measured using a spectrofluorometer with an excitation wavelength of 370 nm and an emission wavelength of 450 nm. Percentage of inhibition was calculated as follows:

$$\left(\%\right)\text{ inhibition }=\;1-\frac{\left(\text{fluorescence of Sample}-\text{Blanko }\right)}{\left(\text{fluorescence of Control}-\text{Blanko}\right)}\;x\;100$$

### 5.8 *In vivo* test

Twenty male Wistar rats, with an average weight of 200 g and age of 8 weeks were divided into 4 groups (n = 5 per group). Three experimental groups were induced to become hyperglycemic with NA as much as 230 mg/kg bw intraperitoneally 15 min before STZ administration (65 mg/kg) in citrate buffer (0.1 M, pH 4.5) after fasting overnight. Nondiabetic control rats were injected with citrate buffer (pH 4.5) only. Diabetic rats with increased fasting blood glucose from 90 to 198 mg/dL were selected for study and divided into 3 groups: diabetic rats, diabetic rats treated with 0.17 mg/mL active fraction from red algae, and diabetic rats with a treatment of 0.255 mg/mL active fraction from red algae. Red algae active fraction was administered every day for 4 weeks. Blood samples were collected from the retro orbital plexus of the rats after overnight fasting using a hematocrit capillary. Rats were fasting overnight before they were euthanized. For examination of gene expression, kidney samples of rats were stored in a tube containing RNA later solution and stored at -80 °C.

### 5.9 Plasma glucose, glycated albumin, and Nε-(carboxymethyl) lysine levels test

Plasma glucose level was determined quantitatively by using the GOD-PAP method using Glucose GOD-PAP Biolab Reagents. Measurement of plasma glucose level was done by spectrophotometry at 500 nm. Plasma GA and CML levels were measured using ELISA method. OD values at 450 nm wavelength were converted to GA and CML levels using CurveExpert software.

### 5.10 Renal RAGE gene expression

Messenger RNA (mRNA) was isolated using RNAprep Pure Kit (Invitrogen) from 0.3 mg of rat kidneys and stored at -80 °C. cDNA was made using iScript ™ cDNA Synthesis Kit with PCR conditions for incubation of 5 min at 25 °C, 30 min at 42 °C, 5 min at 85 °C, and finally at 4 °C, with the number of cycles was 1 cycle. Renal RAGE gene analysis used SsoFast TM EvaGreen ® Supermix, with a PCR conditions of 30 s for enzyme activation at 95 °C, 5 s at 95 °C for denaturation, 5 s at 55 °C for annealing, and finally 2–5 seconds/step at 65–95 °C for melting curve. The number of cycles was 40 cycles using the RAGE primer sequence F: 5 ′CACCATGCCAGCGGGGAC 3′ and R: 5 ′AGCTCTGCACGTTCCTCCTCAT 3′ and the beta actin primer sequence F 5′ TGTCACCAACTGGGACGATA 3′ and R 5′ ACCCTCATAGATGGGCACAG 3’ for reference. The level of gene expression was measured using relative quantitation, the 2^-ΔΔCT^ arithmetic formula (Livak and Schmittgen, 2001).

### 5.11 Statistical analysis

The normality of the data was first analyzed using Saphiro-Wilk test. The normal distributed data then were tested using Paired T test and one-way ANOVA, followed with Duncan post hoc analysis for parametric test. A *p* < 0.05 was considered statistically significant using SPPS 22 (IBM Corporation, Armonk, NY, USA).

## Declarations

### Author contribution statement

E. Yulianti: Conceived and designed the experiments; Performed the experiments; Analyzed and interpreted the data; Contributed reagents, materials, analysis tools or data; Wrote the paper.

Sunarti; M. S. H. Wahyuningsih: Conceived and designed the experiments; Performed the experiments; Analyzed and interpreted the data; Contributed reagents, materials, analysis tools or data.

### Funding statement

This work was supported by faculty of Medicine, Public Health and Nursing, 10.13039/501100012521Universitas Gadjah Mada, Yogyakarta, Indonesia through the scheme of Research Grant for Lecturer-doctoral student of Year (2019).

### Data availability statement

Data will be made available on request.

### Declaration of interests statement

The authors declare no conflict of interest.

### Additional information

No additional information is available for this paper.
